# Characterization of the Compounds Released in the Gaseous Waste Stream during the Slow Pyrolysis of Hemp (*Cannabis sativa* L.)

**DOI:** 10.3390/molecules27092794

**Published:** 2022-04-27

**Authors:** Laetitia Marrot, Kristine Meile, Mariem Zouari, David DeVallance, Anna Sandak, Rene Herrera

**Affiliations:** 1 InnoRenew CoE, Livade 6, 6310 Izola, Slovenia; laetitia.marrot@innorenew.eu (L.M.); mariem.zouari@innorenew.eu (M.Z.); devallance@innorenew.eu (D.D.); anna.sandak@innorenew.eu (A.S.); 2 Andrej Marušič Institute, University of Primorska, Muzejski Trg 2, 6000 Koper, Slovenia; 3 Biorefinery Laboratory, Latvian State Institute of Wood Chemistry, Str. Dzerbenes 27, LV-1006 Riga, Latvia; kristine.meile@kki.lv; 4 Faculty of Mathematics Natural Sciences and Information Technologies, University of Primorska, Muzejski Trg 2, 6000 Koper, Slovenia; 5 Chemical and Environmental Engineering Department, University of the Basque Country (UPV/EHU), Plaza Europa 1, 20018 San Sebastián, Spain

**Keywords:** residual stream, hemp by-products, slow pyrolysis, gaseous phase, thermal decomposition, biocompounds

## Abstract

This study aims to characterize and valorize hemp residual biomass by a slow pyrolysis process. The volatile by-products of hemp carbonization were characterized by several methods (TGA, UV-VIS, TLC, Flash Prep-LC, UHPLC, QTOF-MS) to understand the pyrolysis reaction mechanisms and to identify the chemical products produced during the process. The obtained carbon yield was 29%, generating a gaseous stream composed of phenols and furans which was collected in four temperature ranges (F1 at 20–150 °C, F2 at 150–250 °C, F3 at 250–400 °C and F4 at 400–1000 °C). The obtained liquid fractions were separated into subfractions by flash chromatography. The total phenolic content (TPC) varied depending on the fraction but did not correlate with an increase in temperature or with a decrease in pH value. Compounds present in fractions F1, F3 and F4, being mainly phenolic molecules such as guaiacyl or syringyl derivatives issued from the lignin degradation, exhibit antioxidant capacity. The temperature of the pyrolysis process was positively correlated with detectable phenolic content, which can be explained by the decomposition order of the hemp chemical constituents. A detailed understanding of the chemical composition of pyrolysis products of hemp residuals allows for an assessment of their potential valorization routes and the future economic potential of underutilized biomass.

## 1. Introduction

Hemp is an annual crop cultivated worldwide for applications in various industries such as textile and paper (hemp fibres), food and feed (hemp seeds), construction (hemp shives), and medicine with the extraction of cannabidiol (CBD) from the hemp flowers and leaves [1]. In Western European countries, hemp is mainly cultivated for CBD production, while the whole stem, including fibres and shives, becomes a waste or low-value by-product [2]. Unprocessed hemp stem biomass is then used as a fertilizer or energy source, but these applications do not bring much value to the biomass, which could instead be integrated into a valorization chain and thus fulfil the sustainable circular economy principles [3]. Interest in manufacturing with renewable materials and compounds instead of fossil resources is rising due to the societal demand for biobased products and sustainability awareness.

A promising solution to valorize residual hemp material is thermochemical conversion into biochar (or biocarbon). Biochar is a carbonaceous product obtained from biomass feedstocks heated at elevated temperatures in an oxygen-starved atmosphere [4]. Thanks to its interesting properties, biochar has been investigated for a wide range of low to high-value applications such as soil fertilizer [5], solid fuel and electrical components [6], sensors [7], and components in batteries and supercapacitors [8]. Biochar’s physical and chemical properties can be adapted by changing production process parameters and by choice of feedstock. Biochar can be obtained from several thermochemical transformations, including slow and fast pyrolysis, gasification, hydrothermal carbonization, flash carbonization and torrefaction [9]. These methods differ mainly by final temperatures and heating rates, which will determine the yield and quality (e.g., surface area and pore volume) of biochar and, consequently, its properties and price. Slow pyrolysis, also called conventional carbonization, and hydrothermal carbonization are two of the most efficient biochar conversion technologies which can be used for a wide range of feedstocks [10]. While the quality of biochar generally increases with the temperature of the pyrolysis process, its yield decreases, and the yield of gases increases. Hence, the production of high-quality biochar often generates large amounts of gaseous residues.

Valorization of these residues is needed to improve the efficiency of overall biochar production [11]. For example, Xin et al. [12] studied the economic advantage of coproducing liquid smoke food flavouring along with biofuels within a small-scale fast pyrolysis process. Moreover, the valorization of compounds from thermochemical conversion waste streams allows for an increase in the profitability of the process. Initially, the degradation of hemicelluloses occurs, which leads to the formation of furfurals and acetic acid. When temperatures increase, the lignin degradation process produces phenolic compounds of different molecular weights, as well as acids, alcohols, aldehydes, ketone esters and heterocyclic derivatives. The complex aqueous streams generated from pyrolysis processes contains solubilized organic compounds which could be collected and used for other applications, thus offering economical potential and promoting the transition of farming towards zero waste with a neutral or positive environmental impact. Without a valorization strategy, the waste then becomes an economic burden for thermochemical biorefineries, which need to treat and recycle the effluents. Literature highlights valorization for several industrial sectors, especially foods, cosmetics, and pharmaceutics [13]. Wilson et al. [14] isolated high purity phenol and catechol from aqueous waste streams generated via fast catalytic pyrolysis. Additionally, components from the waste streams presented interesting properties such as antioxidant activity, antimicrobial, antiviral, or anti-allergenic effect, food additives and fragrances [15,16,17,18]. Salami et al. [19] investigated the potential of using slow pyrolysis of hemp shives, roots and leaves, and characterized the liquids retrieved when temperatures were between 275–350 °C. Their research found that the pyrolysis processing of hemp materials resulted in recovered acetic acid, 2,6-dimethoxyphenol, 2-methoxyphenol, and cannabidiol liquids. In a similar study, the resulting distillates of three types of hemp shives heated using slow pyrolysis up to 350 °C were identified to produce baseline data for potential hemp valorization [20]. Barbero-López et al. [21] also investigated distillates from hemp fibre pyrolysis and found that acetic acid was the main chemical constituent. Additionally, the distillates from hemp had higher amounts of methanol, as compared to spruce and birch pyrolyzed under the same conditions.

The objective of this study was to investigate the volatile by-products of hemp carbonization, with the purpose of identifying the possibilities to valorize the chemical products which arise during slow pyrolysis. Additionally, an analytical approach was implemented to better understand the pyrolysis reaction mechanisms. The components were initially separated into fractions according to the range of degradation temperatures. The fractions were isolated, and their phenolic content and potential antioxidant activity were explored and then finally identified through chromatography.

## 2. Results and Discussion

### 2.1. Thermochemical Degradation of Hemp

**Figure 1:**
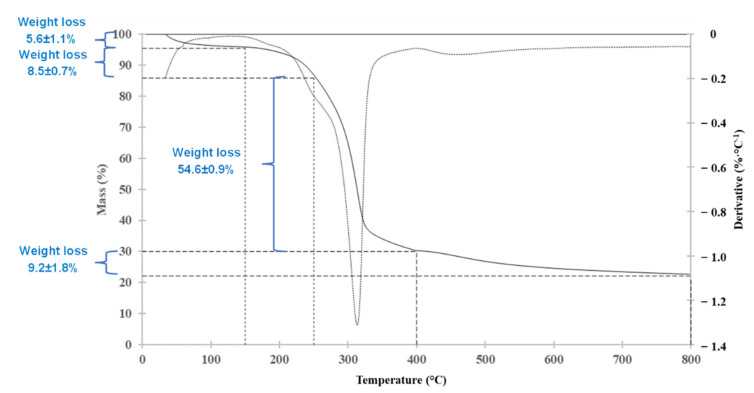
Thermogravimetric degradation of hemp from 0 to 800 °C under N_2_ atmosphere.

Figure 1 shows the thermogravimetric degradation of hemp from 0 to 800 °C under N_2_ atmosphere. Lignin is reported to slowly decompose over a broader temperature range than cellulose and the hemicellulose components of hemp [22], so part of the weight loss of each temperature range includes lignin-related compounds. Between 20 and 150 °C, an endothermic area appears corresponding to moisture and the delicate volatiles (extractives) released, reaching a 5.6% weight loss with a heating rate of 1.67 °C/min. Between 150 and 250 °C, hemicelluloses start to degrade (8.5% out of 24.1% oven-dried basis reported hemicellulose composition) [22,23,24,25], and between 250 and 400 °C, the major mass loss (54.6%) occurs which corresponds to the superposition of the decomposition of the remaining hemicelluloses (about 15.6%), and the decomposition of cellulose (46.1% oven-dried basis reported cellulose composition). The maximum degradation of the basic organic components occurs between 315 °C and 400 °C [23,24,25,26], where an exothermic peak from depolymerization of the hemicelluloses was observed at 320 °C. From 400 to 800 °C, lignin decomposition continues (22.81% oven-dried basis reported lignin composition), and the sum of the mass losses achieved after decomposition corresponds to 77.20%.

After thermochemical conversion (until 1000 °C) in the tube furnace, 57.4 g of solid residue was obtained, which resulted in a 29.2% yield. In comparison, after TGA analysis (up to 800 °C), less solid residue was obtained (22.2 ± 1.8%). The difference between the residual content from thermochemical conversion and from TGA is mainly due to a scale effect and equipment precision [26], the lower scale of analysis in the TGA (few mg), and higher weighing accuracy compared to the amount converted in the tube furnace could generate these differences. Nevertheless, the information provided by the TGA was decisive for the definition of the collected ranges based on the peaks and segments drawn in the thermogravimetric differential.

### 2.2. Characterization of the Slow Pyrolysis Volatile Fractions

Based on the previously discussed TGA results, volatiles arising in the slow pyrolysis process were captured at the exit of the tube furnace as fractions at different temperatures: F1 at 20–150 °C, F2 at 150–250 °C, F3 at 250–400 °C, and F4 at 400–800 °C. The collected fractions underwent a series of characterizations, and the results are summed up in Table 1. The concentration of the non-volatile compounds in the fraction up to 150 °C (F1) was 6–8 times lower than in the subsequent fractions (F2–F4), whose concentrations were relatively similar. These values can be correlated with the weight loss found in the TGA, where the largest mass loss was found in F3, while F2 and F4 present values within the same range. In addition, the acid number (AN) was evaluated as a prediction value of products present in the fractions, such as phenolic acids and light oxygenated organic compounds. Volatile acids, mainly acetic acid, are considered to be potential products that can be recovered from pyrolysis products [25]. The AN increased during the thermochemical conversion, obtaining the highest values from 400–800 °C (F4). However, since the compounds present in the fractions were diluted with the capturing water, the ANs in all the fractions were low (<2) compared with that found in the literature [24,26]. Moreover, the pH range varied from 5.5 to 3.5, which indicates that the concentration of weak organic acids increased during the thermochemical conversion of hemp.

With respect to the total phenolic content (TPC) calculated on each fraction, the order was as follows: F4 > F1 > F3 > F2. These results showed that TPC varied depending on the fraction but did not correlate with the increase in temperature or with the decrease of the pH value, implying that the acidity of the collected fractions was more related to low molecular volatile acids rather than to phenol carboxylic acids. In general, the fraction that is composed mainly of lignin degradation products (F4) showed the highest TPC, while the fraction including the main degradation products from hemicelluloses (F2) had the lowest TPC. Moreover, the most volatile fraction (F1) showed high TPC, although the gravimetric concentration was quite low. The lack of correlation between the gravimetrically determined non-volatiles and the chemically-determined phenols was related to phenol loss while drying at 105 °C. These results agree with those found in previous studies in which significant concentrations of phenol compounds were observed in the volatile fractions of hemp fast pyrolysis (up to 560 °C) [27] and in the hemp slow pyrolysis distillates [20]. The main constituents found include monophenols, phenol derivatives, guaiacols and syringols [28,29].

The antioxidant activity of the fractions was determined by the DPPH assay (IC50), in which the most active fractions need a lower concentration to inhibit 50% of the DPPH radical used in the evaluation. A linear and positive correlation was observed between TPC and DPPH content (R = 0.73), where the fractions with high TPC contributed significantly to the DPPH radical scavenging capacity of the fraction. These preliminary results showed that compounds present in fractions F1, F3, and F4 potentially act as antioxidants, being mainly phenolic molecules such as guaiacyl or syringyl derivatives issued from the lignin degradation [14,15,30,31,32]; however, furans are also reported to show antioxidant properties [15].

**Figure 2:**
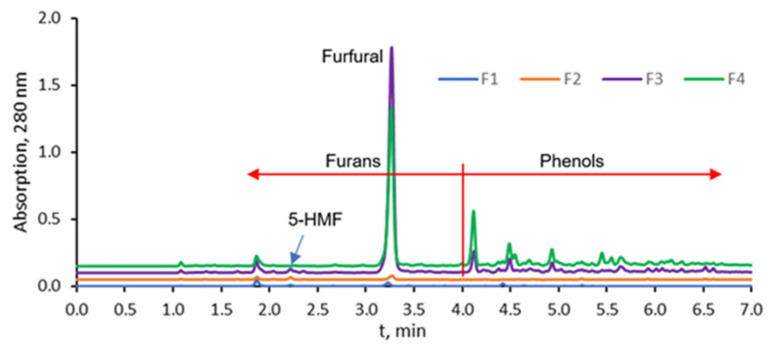
UHPLV-UV chromatograms (λ = 280 nm) of the main fractions of pyrolysis waste streams.

Moreover, UHPLC analysis was performed to acquire more detailed information about the chemical composition of the fractions. Figure 2 shows the UHPLC-UV (λ = 280 nm) chromatograms of the fractions. This analytical method is aimed at determining furan and phenol derivatives but excludes low molecular weight products, such as formic acid, formaldehyde, acetaldehyde, etc. Based on the retention times of the standards, an approximate division between furans and phenols was made at 4 min (Figure 2). Table 2 shows the relative quantitative comparison of these fractions based on the total peak area up to 4 min and after 4 min at 280 nm wavelength. The summed peak areas of furans and phenols showed that fractions F1 and F2 had a very low concentration of constituents (2.2% of the total detected analytes) as compared to F3 and F4, meaning that the yield of condensable furan and phenol products at pyrolysis temperatures up to 250 °C was almost negligible.

The yield of condensable furan and phenol products at higher temperatures was similarly distributed between fractions F3 (47.3%) and F4 (48.3%). However, with increasing temperature, the ratio between furans and phenols changed. Namely, the higher the pyrolysis temperature, the more phenols could be detected in the sample. This is explained by the more easily-occurring thermal decomposition of holocellulose (source of furans), followed by the decomposition of lignin (source of phenols) with more severe pyrolysis treatment. By comparison to standards, peaks with t_R_ 2.22 and 3.28 min were identified as 5-HMF and furfural, respectively. Furfural was the dominant furan in all fractions. The relative concentration of 5-HMF was significantly lower and below the detection limit in F4. Concentration of 5-HMF in F1, F2, and F3 was 0.13, 0.42, and 1.2 µg/mL, respectively. Furfural was 0.74, 0.14, 32, and 15 µg/mL in F1, F2, F3, and F4, respectively. Furfural has been described as a valuable platform chemical obtainable by pyrolysis from different biomass feedstocks [30].

### 2.3. Separation of the Fractions into Subfractions

To expand the characterization and to explore the possibilities of specific chemical compounds production, the fractions collected from the tube furnace were further separated by preparative liquid chromatography. For each fraction, the choice of the most suitable eluent was defined thanks to the thin layer chromatography (TLC) screening. The most effective mobile phase observed with TLC was acetone–ethanol; thus, a stepwise gradient was optimized for the fractionation process from 100:0 to 90:10 (*v*/*v*). A mixture of acetone (solvent A) and ethanol (solvent B) with an increasing gradient ratio was used to separate each fraction into subfractions (Sf) in the preparative chromatography system, based on their affinity with the eluent and on their UV and ELS signals.

Table 3 shows the results of the fractionation according to the ELSD and UV absorbance for the whole UV spectra and for some specific wavelengths (254, 265, 280 and 320 nm). The chemical compounds contained in each fraction have different UV spectra, and thus a different response factor at a specific wavelength. However, it was observed that the sample response corresponding to the wavelength at 280 nm was the closest to the response for the whole UV spectra. Based on these results, F1 was decomposed into four subfractions (Sf-1 to Sf-4), in which Sf-1 and Sf-4 contain only UV-detected groups, and Sf-2 and Sf-3 contain compounds detected by ELSD. F2 was decomposed into three subfractions, all of them separated exclusively by UV signals. Moreover, F3 was divided into three subfractions where Sf-1 contains only UV-detected groups, Sf-2 contains ELSD groups and Sf-3 is a mixture of compounds detected with both UV and ELSD detectors. Fraction 4 was divided into three subfractions where Sf-1 and Sf-3 contain UV detected groups and Sf-2 presents only compounds with ELSD activity.

### 2.4. Qualitative Characterization of the Fractions and Subfractions

Qualitative analysis of the fractions and subfractions was completed by the more sensitive QTOF-MS detection. In LC-MS, the response of a chemical is influenced by the chemical’s structure and chemical qualities and also its interaction with the different parameters of the MS instrument. We observed that in the given conditions used for untargeted MS analysis, some analytes did not ionize at all (furfural), but some produced ions that did not match their molecular ions (5-HMF). However, many aromatic structures were detected and identified.

**Figure 3:**
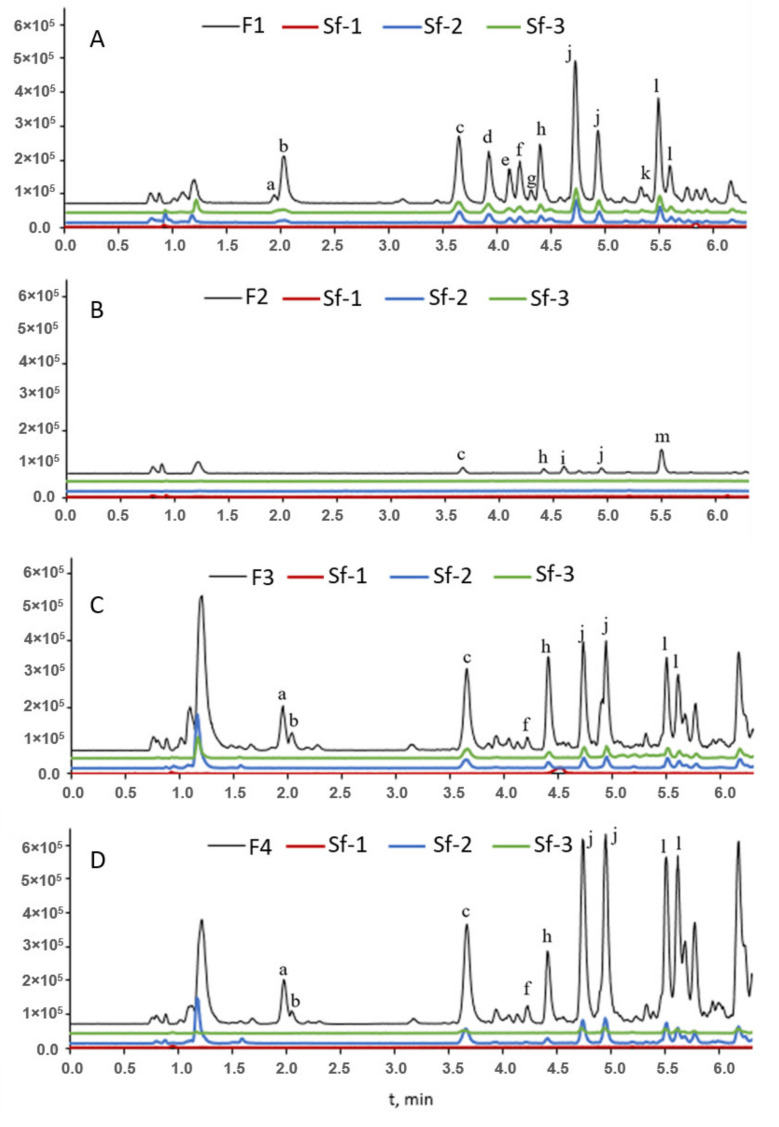
UHPLC-MS chromatograms of the waste stream fractions (**A**–**D**) and their subfractions. Identified structures in each peak: a—C_6_H_10_O_4_; b—C_6_H_6_O_3_; c—C_6_H_6_O_2_; d—C_7_H_6_O_3_; e—C_9_H_8_O_3_; f—C_8_H_8_O_3_; g—C_9_H_8_O_3_; h—C_9_H_10_O_4_; i—C_7_H_6_O_2_; j—C_7_H_8_O_2_; k—C_8_H_8_O_2_; l—C_8_H_10_O_2_; m—C_10_H_10_O_3_.

The formulas of the main structures detected in the fractions F1, F2, F3, and F4 can be cautiously identified as follows. The peak with t_R_ 1.87 min had the most intensive ion 125.03 Da, corresponding to C_6_H_6_O_3_, the molecular structure of methyl furoic acid. Peak with t_R_ 4.12 min and 139.08 Da ion was related to the structure C_7_H_8_O_3_. Peak with t_R_ 4.42 min and 123.05 Da ion fits with C_7_H_8_O_2_ methoxyphenol (guaiacyl) structure. At 4.49 min there was also a structure with a 123.05 Da ion, a fragment of a guaiacol derivative. The other peaks, even though adequately separated by UV detection, were not resolved in the MS chromatograms, because even low concentrations of co-eluting compounds interfered with the identification of peaks after 4.5 min. Namely, there were more detected peaks with MS (Figure 3) than with UV detection, making it difficult to assign a mass spectrum to a peak in the UV chromatogram.

**Figure 4:**
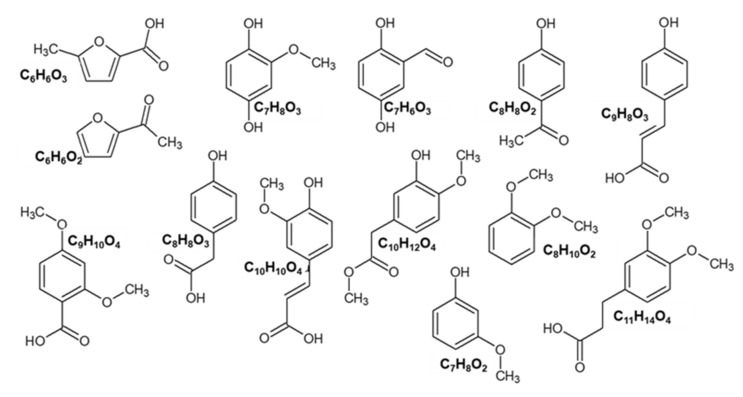
Some of the chemical structures identified from the MS spectra (isomers possible).

The main identified structures, either molecular or fragment ions, are shown in Figure 4. The peaks with earlier elution and ions with mass 125.03 and 109.03 Da were confirmed to be furan derivatives—methyl-furoic acid and acetylfuran. Peaks with later elution were phenols with typical structures, such as methoxyphenol or guaiacol (123.05 Da), dihydroxybenzaldehyde (137.03), dimethoxybenzoic acid (181.04 Da) or other isomers. Figure 3 shows that F3 and F4 were qualitatively very similar. In F1 there was a different ratio between furans a and b and several phenols (peaks d,e,g). These large differences were not evident in F3 and F4.

F1 had a larger number of individual compounds detected both by UV and MS in negative electrospray mode. Figure 3A shows the UHPLC-MS chromatograms of sample F1 and its subfractions. It appears that the subfractions Sf-1 and Sf-2 had a similar qualitative composition as the mother fraction. The main identified chemical formulas in F1 were as follows: C_6_H_6_O_3_ (125.03 Da), C_6_H_6_O_2_ (109.03 Da), C_7_H_6_O_3_ (137.03 Da), C_9_H_8_O_3_ (163.04 Da), C_7_H_8_O_2_ (123.05 Da), C_10_H_10_O_4_ (193.05 Da) and C_8_H_8_O_2_ (135.05 Da). Fewer compounds were detected in F2 (Figure 3B).

The fractions F3 and F4 (Figure 3C,D) had very similar qualitative compositions, with quantitative differences, mostly regarding the ratio between furans and phenols. The main structures identified were C_6_H_6_O_3_ (125.03 Da), C_6_H_6_O_2_ (109.03 Da), C_8_H_8_O_3_ (151.04 Da), C_9_H_10_O_4_ (181.04 Da), C_7_H_8_O_2_ (123.05 Da), C_10_H_12_O_4_ (195.07 Da), C_11_H_14_O_4_ (209.09 Da), C_8_H_10_O_2_ (137.07 Da).

The preparative HPLC separation of the F3 main fraction had results similar to the F1 separation—equal distribution between subfractions Sf-2 and Sf-4. Separation of the F4 main fraction resulted in the subfraction Sf-2 with a similar qualitative composition as the main fraction, while Sf-3 had only a few chemical constituents related to the following structures: C_6_H_6_O_2_ (109.03 Da), C_7_H_8_O_2_ (123.05 Da) and C_8_H_10_O_2_ (137.07 Da).

## 3. Materials and Methods

### 3.1. Hemp

Hemp (*Cannabis sativa* L.) from the Futura 75 variety was grown in 2020 in Frankolovo (Slovenia) and supplied by the Vrhivšek farm. The composition of the hemp stems used in this study was previously assessed [6] as follows: α-cellulose (46.09%), hemi-cellulose (24.12%), Klason lignin (22.81%), total extractive compounds (4.14%) and ash (2.72%), on an oven-dried basis. The stems were stored in a dry environment prior to undergoing thermochemical conversion.

### 3.2. Thermogravimetric Analysis of Raw Hemp

Thermogravimetric analysis (TGA) was performed on 2–5 milligrams of hemp stem with a Waters TA Instrument TGA 5500 Thermogravimetric Analyzer to visualize the degradation range according to the temperature of treatment. The samples were heated under an inert atmosphere (N_2_ flow 25 mL/min) from 20 °C to 400 °C at 1.67 °C/min, and from 400 °C to 800 °C at 10 °C/min. The heating rates were chosen to simulate the conditions of the thermochemical conversion occurring in the tube furnace, identifying the key degradation steps of the process and selecting the fractions to collect according to the degradation of products from hemp [6]. However, while the thermochemical conversion in the tube furnace was continued up to 1000 °C, the TGA maximum temperature was limited to 800 °C so as not to damage the platinum pans containing the samples.

### 3.3. Thermochemical Conversion

**Figure 5:**
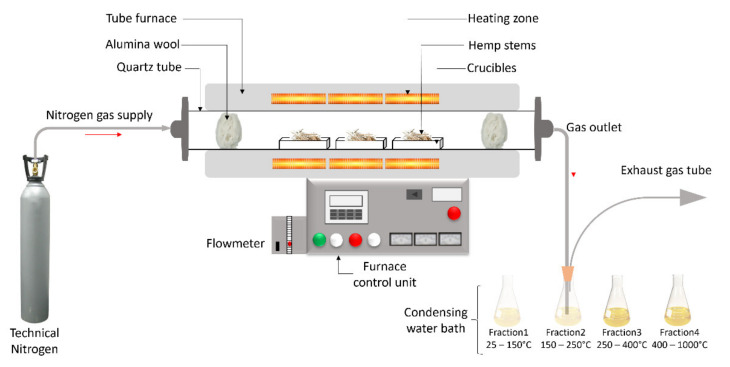
Schematic of the thermochemical conversion process and sampling of the aqueous fractions.

Hemp stems (196.5 g) were cut to smaller pieces of 15 cm length and placed in rectangular alumina boats inside a Nabertherm RSRC 120-1000/13 tube furnace. No drying step was added prior to the thermochemical conversion in order to preserve the sensitive volatiles in the stems. The thermochemical process was performed under an inert N_2_ atmosphere from 20 to 400 °C at 100 °C/h, and from 400 °C to 1000 °C at 600 °C/h. A higher heating rate was chosen for the second part of the conversion to speed up the process, taking into account that most of the volatiles are released at temperatures below 400 °C [6]. While the solid biochar residue remains in the crucibles, the nitrogen flow carries the gaseous compounds out of the tube furnace through a pipe ending in a 500 mL distilled water trap (Figure 5). After the thermochemical conversion cycle, the furnace was cooled, and the solid residues were removed. The solid biochar residue was weighed to calculate the yield based on the following formula (1).

(1) $$Yield\;solid\;residue\;\left(\%\right)=\frac{Mass\;biocarbon\;\left(g\right)}{Mass\;hemp\;biomass\;\left(g\right)}\times 100\;\%$$

### 3.4. Collection of the Waste Streams and Characterization

Four temperature ranges (20–150 °C, 150–250 °C, 250–400 °C and 400–1000 °C) were selected according to the degradation range of the hemp macro components, to collect fractions containing the gaseous products trapped in the water. At the end of each temperature range, the final temperature was maintained for 10 min to give time for the system to evacuate the gas produced during the corresponding step. After each temperature range, the 500 mL of water was collected and replaced with 500 mL of fresh distilled water for the following step.

The non-volatile concentration (mg/g) of each fraction was evaluated by measuring 10 mL in petri dishes, placing them at 105 °C and weighing the residue. The acid number of each fraction was calculated by titration (mg KOH/g), and the pH was measured with an electrochemistry meter [Thermo Scientific] (Waltham, MA, USA).

### 3.5. Total Phenolic Content and Antioxidant Activity of the Fractions

The total phenolic content of each fraction was measured following the Folin–Ciocalteu method [31,32]. First, serial dilutions of gallic acid solutions in distilled water were prepared and used later to draw a calibration curve. Then, 0.3 mL of the solution to be tested (each fraction and gallic acid solutions) was mixed with 2.5 mL of aqueous Folin–Ciocalteu reagent (10% *w*/*v*). Then, the mixtures were covered for 30 min, and the absorbance of all the solutions was measured at 765 nm with a UV-VIS Spectrophotometer UV7 [Mettler Toledo] (Columbus, OH, USA), using methanol as a blank. The amount of total phenolic compounds was expressed as mg/g of gallic acid equivalents in milligrams per gram (mg GAE/g) of dry extract. The experiment was repeated in triplicate, and the mean value was reported.

The antioxidant activity of the butylated hydroxytoluene (BHT) standard and the fractions was evaluated by comparing their radical scavenging activity to the one of the stable 1,1-diphenyl-2 picrylhydrazyl (DPPH) by DPPH modified method [31,33]. The first 3 mL of DPPH solution (0.1 mM) was mixed with 3 mL of sample (BHT or fraction). The mixtures were stored in a dark environment for 30 min at room temperature. After this time, the absorbance of all the solutions was measured at 517 nm with a UV-VIS Spectrophotometer UV7 [Mettler Toledo], using the methanol-based DPPH solution as control and methanol or water as blank. The percentage inhibition of DPPH activity was calculated using the formula (2):

(2) $$Inhibition\;of\;DPPH\;activity\;\left(\%\right)=\frac{A-B}{A}\times 100\%$$

where, *A* is the absorbance of the control and *B* is the absorbance of the sample.

### 3.6. Separation of the Water-Soluble Compounds into Subfractions

With the view toward separating each fraction into subfractions based on the active groups they contain, thin-layer chromatography (TLC) was used preliminarily to determine suitable eluents (solvents and ratios). TLC is a quick, sensitive, and inexpensive technique that helps to determine the number of components in a mixture and the solvent composition for preparative chromatographic separations [34].

The samples were prepared by spotting an aliquot with a capillary on the TLC plate and then placing it in a glass container containing enough organic solvents to activate the interaction between the two phases (mobile phase and stationary phase). Different apolar-to-polar organic solvents (hexane-methanol-methylene and chloride-ethanol) were used and combined to find the best mixture separation. The TLCs with the best separations were visualized by UV light and used as the starting point for the chromatographic separation.

Before running the separation system, the water-insoluble particles were removed from the fractions by filtering with a Syringe Filter (CA, pore size 0.45 µm). Then the obtained fractions were separated into subfractions according to their polarity and affinity. Flash and preparative chromatography was carried out using a Buchi Pure C-850 Flashprep chromatography system equipped with a Sepacore pump and UV and ELSD detectors. FlashPure EcoFlex spherical silica cartridge column (4 g, C18, 50 µm) with a 15 mL/min flow rate was used. The eluents were acetone (Eluent A) and ethanol (Eluent B), and different gradients and exposure times were explored [35]. The chromatography system allows the determination of the UV absorbance and the Evaporative Light Scattering Detection (ELSD) of the fractions being analysed and the proceeding to the separation of the subfractions accordingly. The UV wavelengths selected for the separation were 240 nm, 285 nm, 310 nm and 330 nm UV with a detector sensitivity fixed at 0.02 AU and the ELSD at 3 mV. The subfractions were then collected and stored in closed vials in a refrigerator at 4 °C until further analysis.

### 3.7. UHPLC Analysis of the Water-Soluble Compounds in the Subfractions

Fractions and subfractions were filtered without dilution through syringe filters (0.45 µm), and 2 µL were injected into the Waters Acquity H-Class UHPLC system, which included an HSS C18 column (2.1 mm × 50 mm, 1.8 µm) at 30 °C and gradient conditions. The gradient consisted of phase A—water with 0.1% formic acid and B—acetonitrile with 0.25 mL/min flow rate. Phase B was held at 10% for 0.5 min, then increased to 90% at 7.0 min and kept there for 0.5 min, returning to 10% at 8.0 min equilibrated for two more minutes. For retention time comparison, the following standards were used—5-hydroxymethylfurfural (≥99%, Sigma-Aldrich) (Ljubljana, Slovenia), furfural (99%, Sigma-Aldrich), vanillic acid (97%, Sigma-Aldrich), syringic acid (≥95%, Sigma-Aldrich), vanillin (99%, Sigma-Aldrich), syringaldehyde (98%, Sigma-Aldrich). For detection, a Waters PDA detector was used at 280 nm wavelength. For untargeted QTOF-MS analysis with a Waters Synapt G-2s mass spectrometer negative electrospray ionization (ESI) was used. The mass spectrometry parameters were set to a trapped collision energy of 6 V, a ramp trap collision energy 15 to 14 V, and a cone voltage 40 V. Chemspider [36] database was used for identification.

## 4. Conclusions

The gaseous waste stream released during the slow pyrolysis of hemp was evaluated as a sustainable way to convert this residual fraction of the process into a feasible source of interesting bioactive compounds. The carbon yield obtained in the slow pyrolysis of hemp was 29%, generating a gaseous stream composed of phenols and furans, which was collected in four temperature ranges (25–150 °C, 150–250 °C, 250–400 °C and 400–800 °C) according to the results of the thermogravimetric analysis. The obtained liquid fractions (F1, F2, F3, F4) were separated into subfractions by flash chromatography using an optimized gradient of acetone–ethanol from 100:0 to 90:10 (*v*/*v*) and obtaining thirteen subfractions with different compounds and concentrations separated by UV and ELSD detectors. The gravimetric concentration up to 150 °C was quite low due to the volatility of the compounds released in that range; however, the fractions obtained from 150 °C to 800 °C presented higher concentrations from 0.24 to 0.34 mg/g. The F1, F3 and F4 showed potential antioxidant capacity and high TPC, while the fraction including the main degradation products from hemicelluloses (F2) presented the lowest values. The detected chemical compound occurrence was in line with the decomposition order of the hemp constituents, with holocellulose releasing furans at lower temperatures, and phenols from lignin mostly at higher temperatures. The yields of the individual chemicals (5-hydroxymethylfurfural, furfural, acetic acid, guaiacol and other aromatic compounds) were low. However, considering the small size of the experimental setup, scaling-up the carbonization process would increase the recovered chemical amount, even for those found in lower levels. Overall, the collection and separation of the compounds produced during the pyrolysis of hemp to obtain biochar appear to have the potential for valorizing the use of waste hemp materials. Further research is warranted to investigate scale-up potential and resulting chemical compounds for different hemp species.

## Figures and Tables

**Table 1:** General analysis results for the slow pyrolysis volatile product fractions.

Parameter	F1	F2	F3	F4
Temperature range	20–150 °C	150–250 °C	250–400 °C	400–800 °C
pH	5.48	4.59	3.47	3.72
Acid number, mg KOH/g	0.136	0.364	0.815	1.148
Non-volatile concentration, mg/g	0.041	0.277	0.344	0.244
TPC, μg GAE/mg sample	1315.00	65.68	1027.99	1833.08
IC_50_ (DPPH), ug/mL	2.951	40.593	3.155	2.121

**Table 2:** UHPLC-UV analysis for the slow pyrolysis volatile product fractions.

Compounds	F1	F2	F3	F4
5-HMF, µg/g	0.13	0.42	1.2	n
Furfural, µg/g	0.74	0.14	32	15
Furan (1.87 min *), rel. units	0.14	0.047	0.24	0.23
Furans (<4 min *), rel. units	0.31	0.30	7.0	5.0
Phenols (>4 min *), rel. units	0.12	0.12	2.1	4.4
Phenol (4.12 min *), rel. units	n	n	0.40	1.0
Phenol (4.42 min *), rel. units	0.05	n	0.03	0.05
Phenol (4.49 min *), rel. units	n	n	0.20	0.34
Phenol (4.54 min *), rel. units	n	n	n	0.17
Phenol (4.93 min *) rel. units	n	n	0.17	0.28
Phenol (5.45 min *), rel. units	n	n	n	0.19
Phenol (5.55 min *) rel. units	n	n	n	0.090

* Retention time in UHPLC-UV chromatograms (Figure 2).

**Table 3:** Description of the subfractions obtained by preparative chromatography.

Subfractions/Fractions	Sf-1	Sf-2	Sf-3	Sf-4
Solvent ratio (A:B)	100:0 → 99:1	99:1 → 98:2	98:2 → 97:3	97:3 → 90:10
F1	Contains 1 group (UV-detected)	Contains mixture groups (ELSD-UV-detected)	Contains mixture groups (ELSD-UV-detected)	Contains 1 group (UV-detected)
F2	Contains 1 group (UV-detected)	Contains 1 group (UV-detected)	Contains 1 group (UV-detected)	-
F3	Contains 1 group (UV-detected)	Contains 1 group (ELSD-detected)	Contains mixture groups (ELSD-UV-detected)	-
F4	Contains 1 group (UV-detected)	Contains 1 group (ELSD-detected)	Contains 1 group (UV-detected)	-

## Data Availability

Not applicable.

## Sample Availability

Samples of the compounds are available from the authors.
